# A Rare Occurrence of Rotational Retro-Esophageal Gastric Body Herniation Through a Nissen Fundoplication

**DOI:** 10.7759/cureus.27732

**Published:** 2022-08-06

**Authors:** Joyce Lin, Vatche Melkonian, Raymond I Okeke, Joseph Platz, Keith S Naunheim

**Affiliations:** 1 General Surgery, Saint Louis University School of Medicine, Saint Louis, USA; 2 Surgery, Saint Louis University Hospital, Saint Louis, USA; 3 Thoracic Surgery, Saint Louis University Hospital, Saint Louis, USA

**Keywords:** stomach herniation, herniation, retroesophageal, gastric body, laparoscopic nissen’s fundoplication

## Abstract

Anti-reflux procedures have become a mainstay in managing gastroesophageal reflux disease (GERD) and hiatal hernia. Unfortunately, post-operative events such as breakdown of the wrap, downward slippage, or transdiaphragmatic herniation of an intact wrap cause these procedures to fail and create complications such as recurrent hiatal hernia and reflux dysphagia, regurgitation, and obstruction requiring revision surgery. We discuss a case of a rotational retro-esophageal herniation of the gastric body through a Nissen fundoplication presenting as obstruction, dysphagia, and regurgitation, highlighting the peculiar nature of this presentation and the ease of misdiagnosis given its rarity.

## Introduction

Laparoscopic Nissen fundoplication is one of the most common anti-reflux procedures for surgical management of gastroesophageal reflux disease (GERD) and hiatal hernia. However, published failure rates vary widely, ranging from 3-30%, and these patients often require revision surgeries [1]. Anatomic causes of failed fundoplication include a partial or complete breakdown of the wrap with or without recurrent hiatal hernia, transdiaphragmatic herniation of an intact wrap, and slippage of the wrap down onto the gastric body, the so-called "slipped wrap" [1-3]. We present a case of a 54-year-old male with GERD and hiatal hernia, managed with Nissen fundoplication, who subsequently developed an acute presentation retro-esophageal gastric herniation/rotation 4.5 months after his initial procedure. This rotational herniation of the proximal stomach behind the esophagus is a phenomenon that has not been well documented in the literature.

## Case presentation

**Figure 1:**
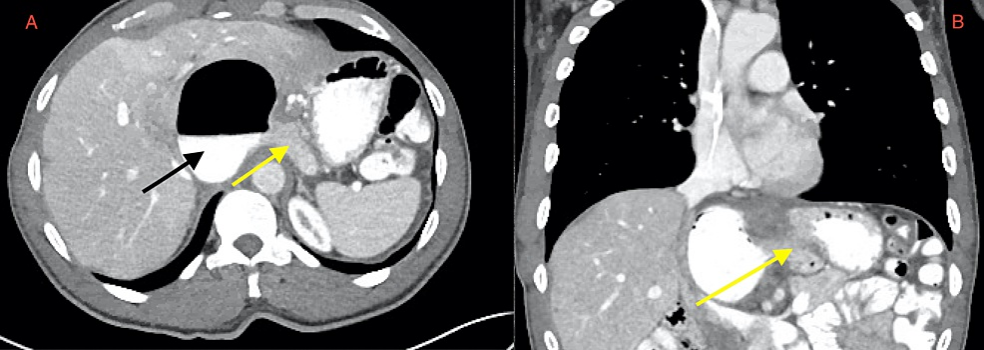
Yellow arrows show herniation of the stomach across the midline in the axial view (A) and the sagittal view (B). Subhepatic air/fluid collection (black arrow) later found to be in the gastric body

**Figure 2:**
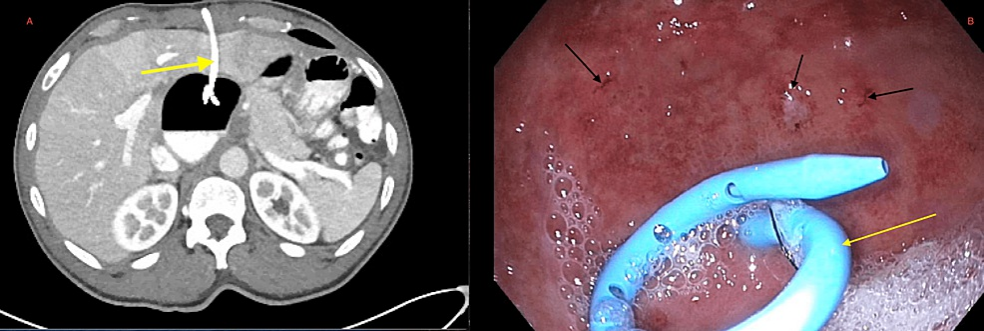
Yellow arrows show the percutaneous drain visualized in the gastric body on CT scan (A) and on the EGD (B). Gastric body ulcers are highlighted by black arrows (B). EGD: esophagogastroduodenoscopy

A 54-year-old male with hiatal hernia and medically refractory GERD underwent an uncomplicated laparoscopic Nissen fundoplication and was asymptomatic for four months. He then presented with a two-week history of sudden onset of epigastric pain, dysphagia, and intermittent regurgitation of food. These symptoms gradually worsened over two weeks until he presented to a healthcare institution where a computer tomography (CT) scan demonstrated an air/fluid collection underneath the left hepatic lobe, immediately lateral and to the right to the wrap (Figure 1). The presumptive diagnosis was a contained gastric perforation. A percutaneous drain was placed transhepatically, which yielded air and yellowish, nonpurulent fluid (Figure 2). The patient was started on total parenteral nutrition (TPN) and intravenous (IV) antibiotics. He was then transferred to our facility for further care. 

On arrival, the patient was hemodynamically stable and in no acute distress. His pain had improved slightly after the drain placement, but he still reported an inability to tolerate oral intake. His physical examination was significant for tenderness in the epigastric region but was otherwise unremarkable. His white blood cell count, hemoglobin level, and electrolytes were all within normal limits. We reviewed the imaging, suspecting that this was not an abscess but the stomach's body that had rotated axially behind the esophagus. 

We took the patient to the operating room, where, via a laparoscopic approach, we elevated the left lobe of the liver and noted that the transhepatic drain was entering a large bubble of the stomach. This part of the stomach was the gastric body rotated behind the wrap into a subhepatic position to the right of the wrap. The stomach was dissected entirely off the underside of the liver, and we removed the percutaneous drain clearly visualized on the esophagogastroduodenoscopy (EGD) (Figure 2B) before the start of the case. We then stapled the drain hole in the gastric wall shut and took down the Nissen fundoplication. We then rotated the body of the stomach through the retro-esophageal window so that it lay in its native location in front of the spleen. We advanced a 56 French dilator into the stomach and performed a Toupet fundoplication. An esophagram was performed on postoperative Day 1, revealing no leakage and the free passage of contrast into the stomach. The patient started a clear liquid diet and was slowly advanced to modified swallowing "minced and moist" diet before discharge on postoperative Day 5.

## Discussion

This case highlights an uncommon complication of the Nissen fundoplication. We identified a rotational retro-esophageal herniation of the gastric body, which acted essentially as an internal hernia, resulting in gastric body distension and functional gastroesophageal obstruction. This complication has not been well documented in the literature. Consequently, there is limited guidance regarding the presentation and management. In 2007, Morgenthal et al. published a case of a 73-year-old female who experienced eight months of nausea and vomiting after her laparoscopic Nissen fundoplication [4]. She then presented with three days of severe epigastric pain and dysphagia and was found to have a slipped fundoplication with retro-esophageal herniation of the stomach, as described in our patient care. Common to these two cases is the abrupt change in symptomatology in patients with this complication, despite initially experiencing minimal or chronic but tolerable symptoms in the months following the initial procedure. Recognition of such change in symptoms would ideally lead to an aggressive diagnostic workup and intervention as this herniation causes proximal gastric obstruction, preventing adequate oral nutrition, and the risk of ischemia as seen in the EGD on our patient. Unfortunately, definitive care was delayed in our patient due to his misdiagnosis until he transferred to our facility. As a result, he had a percutaneous drain placed due to the incorrect assumption that this was a gastric perforation rather than gastric herniation. Fortunately, this drain was functionally a decompressive gastrostomy tube which led to temporary improvement of symptoms and perhaps prevented further complications during the time required for transfer and eventual definitive treatment.

Long-term post-fundoplication complications which result in recurrent reflux, dysphagia, and regurgitation occur in up to 30% of patients [3]. When such symptoms arise and prove unresponsive to conservative measures, the surgeon should undertake a complete anatomic and functional evaluation, including barium swallow, chest/upper abdomen CT with oral contrast, upper endoscopy, 24-hour pH evaluation, esophageal manometry, and gastric emptying scan. The performance of such a complete workup allows for a thorough understanding of the underlying problem's anatomy and physiology. We managed the patient in this case as we would have with any other failed fundoplication: the takedown of the prior fundoplication and revision surgery. A 2010 systematic review by Van Beek et al. found that laparoscopic redo fundoplication effectively reduced symptoms and improved outcomes after failed fundoplication [1]. Some authors suggest that revision surgery with a Toupet fundoplication is the preferred method, especially in patients with an element of post-op dysphagia, such as in this case [5-6]. Post-op dysphagia is often due to structural complications, which include transdiaphragmatic herniation of part or all the wrap or herniation of the stomach proximal to a slipped wrap [1-3]. Although slipped wraps are not the most common structural cause of failed fundoplication, they have been documented in the literature and identified after Nissen procedures [1, 7-8], not Toupet procedures. 

Toupet fundoplication was previously thought to have an increased rate of GERD symptom recurrence compared to Nissen fundoplication [9-10]. Therefore, it was considered advisable in patients with significant pre-op esophageal dysmotility and dysphagia without GERD [11]. However, more recent studies have shown no difference in GERD symptom control or patient satisfaction after both methods [12-16]. Furthermore, Nissen fundoplication is associated with more significant side effects, including post-op dysphagia [12-15], even in the absence of pre-operative esophageal dysmotility [12]. Toupet fundoplication is the preferred anti-reflux surgery for some surgeons who believe it yields similar symptom control with decreased incidence of postoperative dysphagia requiring revision [13]. However, surgeons perform a greater number of Nissen fundoplications, and its side effects and complications have been studied more. Regardless of the most appropriate choice of fundoplication, the crux of the discussion is the awareness of possible complications from these procedures and the high index of suspicion in the postoperative period.

## Conclusions

Clinicians should consider uncommon presentations of common complications from anti-reflux procedures when patients present with dysphagia, poor oral intake, or increased GERD symptoms. Workup should be thorough and include anatomic and physiologic testing, maximizing the probability of obtaining the correct diagnosis and providing appropriate treatment.
